# E2F5 Overexpression in Laryngeal Squamous Cell Carcinoma: Associations With Neutrophil Extracellular Traps in the Tumor Microenvironment

**DOI:** 10.14740/wjon2610

**Published:** 2025-12-17

**Authors:** Ke Jun Wu, Feng Zhao, Yu Feng Li, Yu Chen, Jia Ying Wen, Di Yuan Qin, Rong Quan He, Li Xiao, Dong Ming Li, Bin Li, Qi Li, Ming Jie Li, Gang Chen, Yi Wu Dang, Jia Shu Jiang

**Affiliations:** a Department of Pathology, The First Affiliated Hospital of Guangxi Medical University, Nanning, Guangxi Zhuang Autonomous Region 530021, China; b Department of Otolaryngology, Head and Neck Surgery, The First Affiliated Hospital of Guangxi Medical University, Nanning, China; c Department of Pathology, People’s Hospital of Lingshan County, Qinzhou, Guangxi Zhuang Autonomous Region, China; d Department of Radiotherapy, The Second Affiliated Hospital of Guangxi Medical University, Nanning, Guangxi Zhuang Autonomous Region, China; e School of Information and Management, Guangxi Medical University, Nanning, Guangxi Zhuang Autonomous Region 530021, China; f Department of Medical Oncology, The First Affiliated Hospital of Guangxi Medical University, Nanning, Guangxi Zhuang Autonomous Region 530021, China; g International Cooperation and External Exchange Department, The First Affiliated Hospital of Guangxi Medical University, Nanning, Guangxi Zhuang Autonomous Region 530021, China; h These authors contributed equally to this study.

**Keywords:** Laryngeal squamous cell carcinoma, E2F5, Neutrophil extracellular traps, Tumor microenvironment, Single-cell RNA sequencing

## Abstract

**Background:**

Laryngeal squamous cell carcinoma (LSCC) is a common malignant tumor of the head and neck, associated with smoking and excessive alcohol consumption. The objective was to investigate the expression pattern of E2F transcription factor 5 (E2F5) in LSCC and its association with neutrophil extracellular traps (NETs), elucidating its role in the tumor microenvironment.

**Methods:**

At the cellular level, single-cell RNA sequencing (scRNA-seq) was employed to analyze the expression of E2F5 and NETs-related genes (S100A8, S100A9, LCN2, etc.). At the tissue level, spatial transcriptomics (ST) was used to examine the E2F5 expression pattern. At the mRNA level, E2F5 expression was assessed through mRNA expression profiling, and at the protein level, expression was validated using immunohistochemistry (IHC) on tissue specimens, including 10 LSCC cases (laryngeal, hypopharyngeal, and oropharyngeal squamous cell carcinomas) and 10 non-LSCC controls (benign lesions such as mucoceles, hemangiomas, and polyps). Clustered regularly interspaced short palindromic repeats (CRISPR) knockout screening combined with the CERES algorithm was utilized to evaluate the impact of E2F5 on LSCC cell line proliferation, with negative/positive dependency scores indicating suppression/promotion of growth, respectively. Single-sample Gene Set Enrichment Analysis (ssGSEA) was used to analyze the correlation between E2F5 and immune cells, and chromatin immunoprecipitation sequencing (ChIP-seq) was performed to validate the transcriptional regulation of NETs-related genes by E2F5. Statistical analyses included Wilcoxon, standardized mean difference (SMD), receiver operating characteristic (ROC), and summary receiver operating characteristic (sROC).

**Results:**

E2F5 exhibited high expression in LSCC epithelial cells and tissues, with elevated expression at both mRNA and protein levels (SMD = 0.24, 95% confidence interval (CI) = 0.0309 - 0.448, sROC area under the curve (AUC) = 0.71, IHC P = 7.2 × 10^-6^, ROC AUC = 1). Knockdown of E2F5 significantly inhibited proliferation in LSCC cell lines (e.g., BICR31, BICR16) (inhibition score < 0). High E2F5 expression was positively correlated with T-helper cells and natural killer (NK) CD56bright cells (R = 0.251, 0.175, P < 0.05) and negatively correlated with neutrophils and Th17 cells (R = -0.293, -0.260, P < 0.05). Cellular and tissue-level analyses revealed high NETs expression in LSCC, with E2F5 also highly expressed in NETs-related cells and regions. ChIP-seq analysis confirmed that E2F5 regulates NETs-related genes. Functional enrichment analysis indicated that E2F5-related genes are involved in transcriptional regulation, chromatin organization, and immune regulation.

**Conclusion:**

E2F5 is highly expressed in LSCC and is associated with the regulation of NETs-related genes. It may contribute to tumor proliferation and immune evasion by reshaping the tumor microenvironment, highlighting E2F5 as a potential therapeutic target that warrants further functional validation.

## Introduction

Laryngeal squamous cell carcinoma (LSCC), one of the most prevalent malignant tumors among head and neck squamous cell carcinomas (HNSCC), originates from the mucosal epithelium of the larynx and is strongly associated with smoking, excessive alcohol consumption, and human papillomavirus infection [1, 2]. Despite advances in diagnosis and treatment, LSCC remains highly invasive with frequent recurrence and poor prognosis [3, 4], underscoring the need to further elucidate its molecular mechanisms and tumor microenvironment to guide precision therapy. The rapid development of technologies such as single-cell RNA sequencing (scRNA-seq), spatial transcriptomics (ST), and integrative multi-omics analyses has provided novel perspectives for uncovering the cellular composition and molecular regulatory networks of LSCC.

In the realm of cancer molecular regulation, the transcription factor E2F5, a member of the E2F family, has increasingly garnered attention. The E2F family plays a central role in regulating critical biological processes, including cell cycle progression [5], DNA replication [6], and apoptosis [7]. Aberrant expression of E2F family members is associated with the development and progression of various cancers. However, the expression pattern and functional role of E2F5 in LSCC have not yet been systematically investigated. Given the demonstrated potential of E2F5 in regulating tumor proliferation and the microenvironment in other cancers, exploring its role in LSCC may provide novel insights into the molecular mechanisms underlying this disease.

Within the tumor microenvironment, neutrophil extracellular traps (NETs) have emerged as a critical immune component, attracting significant interest in cancer research in recent years. NETs are web-like structures released by neutrophils, enriched with hallmark molecules such as S100A8, S100A9, and LCN2. Studies indicate that NETs not only contribute to antibacterial defense but may also exacerbate tumor progression by promoting inflammatory responses [8], immune evasion [9], and tumor cell invasion [10]. For instance, research has shown that S100A8/A9 amplifies neuroinflammatory responses in traumatic brain injury by enhancing NETs formation [11]. Additionally, in liver disease-related studies, LCN2 has been shown to be expressed in NETs, enhancing antibacterial capacity through synergistic interactions between neutrophils and hepatocytes [12]. In LSCC, NETs may promote tumor cell proliferation and invasion by releasing pro-inflammatory factors and modulating the expression of tumor microenvironment-related genes. As a transcription factor, E2F5 may participate in shaping the LSCC tumor microenvironment by directly or indirectly regulating the expression of NETs hallmark genes (e.g., S100A8/A9, LCN2), thereby influencing tumor progression. However, the specific molecular mechanisms and potential synergistic interactions between E2F5 and NETs in LSCC remain to be elucidated.

This study integrates scRNA-seq, ST, mRNA profiling, and immunohistochemistry (IHC) to systematically investigate E2F5 expression in LSCC and its association with NETs. We employ a multimodal strategy: scRNA-seq and ST capture cellular and tissue-level patterns but are limited by dissociation bias and spatial resolution; bulk RNA profiling and IHC provide cross-cohort and protein-level validation; clustered regularly interspaced short palindromic repeats (CRISPR)-CERES assesses functional dependency, while single-sample Gene Set Enrichment Analysis (ssGSEA) and ChIP-seq link E2F5 to immune infiltration and transcriptional regulation of NETs-related genes.

## Materials and Methods

### scRNA-seq analysis of E2F5 in LSCC

scRNA-seq analysis of E2F5 utilized LSCC-related scRNA datasets (GSM8002074, GSM8002075, GSM8002076, from GSE252490), accessible via the Gene Expression Omnibus (GEO) database. Data preprocessing and filtering were performed using the “Seurat” package [13], removing genes expressed in fewer than three cells and cells expressing fewer than 50 genes. Quality control retained cells with gene expression counts > 500 and mitochondrial content < 25%. After data normalization, principal component analysis (PCA) was conducted for dimensionality reduction, selecting the top 20 principal components for clustering analysis. Secondary dimensionality reduction was performed using the uniform manifold approximation and projection (UMAP) algorithm. LSCC-related epithelial cells were identified using KRT5, KRT14, TP63, and CDKN2A markers, while NETs-related cells were identified using S100A8, FUT4, LCN2, S100A9, and HSP3A markers.

### Spatial transcriptomics analysis of E2F5 in LSCC

LSCC-related scRNA-seq were obtained from the GEO database under the series accession number GSE252490. This dataset included three individual samples: GSM8002074, GSM8002075, and GSM8002076. Data were processed using the “Seurat” package, with normalization performed via the SCTransform method to standardize the dataset, specifying spatial data usage and disabling verbose output to simplify computation. The SpatialDimPlot function generated spatial dimension figures, visualizing cell spatial distribution in samples. LSCC tissue regions were identified using KRT5, KRT14, TP63, and CDKN2A markers. Cell spatial locations were visualized alongside E2F5 expression patterns, and NETs-related tissue regions were identified using S100A8, FUT4, LCN2, S100A9, and HSP3A markers.

### E2F5 mRNA expression profiling in LSCC tissue samples

To investigate E2F5 mRNA expression differences between LSCC and non-LSCC samples, E2F5 expression data were collected from multiple databases, including GEO, ICGC, GTEx, SRA, TCGA, PubMed, and ArrayExpress. The search formula was: ((laryngeal OR laryngeal squamous cell carcinoma OR LSCC OR HNSCC OR SCC OR carcinoma of larynx OR kehlkopfkrebs OR laryngopharynx) AND (tumor OR neoplasm OR phyma OR malignancy OR malignant OR malignance OR cancer OR carcinoma OR carcinosis)). Clear inclusion and exclusion criteria were established: 1) only human primary LSCC tissues were analyzed; 2) experimental cohorts included LSCC diagnostic tissue samples, with normal tissue samples as controls; 3) both experimental and control groups required ≥ 3 samples. Exclusion criteria applied to datasets with < 3 samples, unreported E2F5 expression, or inclusion of metastatic or recurrent LSCC tissues. Datasets from the same GEO platform were merged into a comprehensive matrix during data preparation. E2F5 mRNA expression levels were normalized and log-transformed using the log2(x + 1) method. Batch effects were corrected using statistical methods from R packages “limma” and “sva”. The meta package (version 4.18-2) calculated standardized mean difference (SMD) for LSCC genes, assessing differences between LSCC and non-LSCC samples to reveal E2F5’s potential pathogenic molecular role in LSCC. Criteria for identifying highly expressed genes in LSCC samples were: 1) gene appeared in ≥ 3 independent studies; 2) SMD > 0; 3) 95% confidence interval (CI) excluded 0. Spearman correlation analysis identified E2F5-related co-expressed genes (CEGs), selected based on: 1) co-expression with E2F5 in ≥ 10 studies; 2) Spearman correlation coefficient r > 0.30; 3) P < 0.05. The clusterProfiler package performed functional annotation of intersecting genes, with Gene Ontology (GO) and Kyoto Encyclopedia of Genes and Genomes (KEGG) pathway analyses exploring involved biological processes and signaling pathways. Genes with NETs-related scores > 1.5, retrieved via gencard for “NETs” or “Neutrophil Extracellular Traps,” were included.

### IHC staining of internal institutional samples

The study collected 20 tissue samples from the People’s Hospital of Lingshan County, including 10 LSCC tissues (laryngeal, hypopharyngeal, and oropharyngeal squamous cell carcinomas) and 10 non-LSCC control tissues (benign lesions such as mucoceles, hemangiomas, and polyps). IHC staining followed strict standard procedures using E2F5 polyclonal antibody (abs113308, absin™, 1:1,000) [14]. Post-staining, tissue microarrays were reincubated, stained, dehydrated, and sealed at room temperature. Staining intensity was graded as: 0 (no staining, blue), 1 (weak staining, light yellow), 2 (moderate staining, yellow-brown), and 3 (strong staining, dark brown). Positive cell proportion was scored as: 0 (< 5%), 1 (5-25%), 2 (26-50%), 3 (51-75%), and 4 (> 75%). The final IHC score was calculated by multiplying intensity and percentage scores (range 0 - 12). Two pathologists independently assessed IHC scores using this composite system and performed Wilcoxon rank-sum tests between LSCC and adjacent non-cancerous tissues. The study was approved by the Medical Ethics Committee (MEC) of the First Affiliated Hospital of Guangxi Medical University (approval No. 2024-S621-01).

### Role of E2F5 in regulating LSCC proliferation

CRISPR screening data were obtained from the DepMap database. To investigate E2F5’s role in LSCC cells, CRISPR knockout screening was employed. The CERES algorithm calculated dependency scores to determine E2F5’s criticality in LSCC cell lines. Negative dependency scores indicated that E2F5 knockout hindered cell line growth, supporting E2F5’s functional necessity in LSCC [15].

### Role of E2F5 in immune cell interactions in LSCC

Spearman correlation analysis was performed to evaluate the role of E2F5 (ENSG00000133740.11) in immune cell interactions in LSCC. Immune cell infiltration was assessed using the ssGSEA algorithm implemented in the GSVA R package (version 1.46.0), with 24 immune cell-specific marker gene sets derived from the Immunity publication [16].

### E2F5 ChIP-seq data

E2F5-related ChIP-seq data analysis utilized public datasets from the Cistrome database [17]. Data were sourced from the ENCODE3 project (ID: ENCSR709DRM_1, CistromeDB ID: 64302), involving Homo sapiens. Targeting E2F5 as the transcription factor, the dataset was generated via ChIP-seq [18]. Data processing followed Cistrome’s standard workflow to analyze E2F5 binding peaks in regulatory regions of NETs-related genes (e.g., S100A8, S100A9, LCN2), validating E2F5’s transcriptional regulatory role in LSCC.

### Statistical analysis

To assess E2F5 protein expression differences in LSCC, the Wilcoxon rank-sum test was used, with P < 0.05 as the significance criterion. A fixed-effects model calculated SMD when heterogeneity was low (I^2^ < 50%); otherwise, a random-effects model was applied. The “pROC” package plotted ROC curves, and STATA 18.0 generated sROC curves. AUC measured E2F5 expression levels, with larger AUC indicating higher expression. The Begg test evaluated publication bias, with P > 0.05 indicating no bias.

## Results

### High E2F5 expression in LSCC

**Figure 1:**
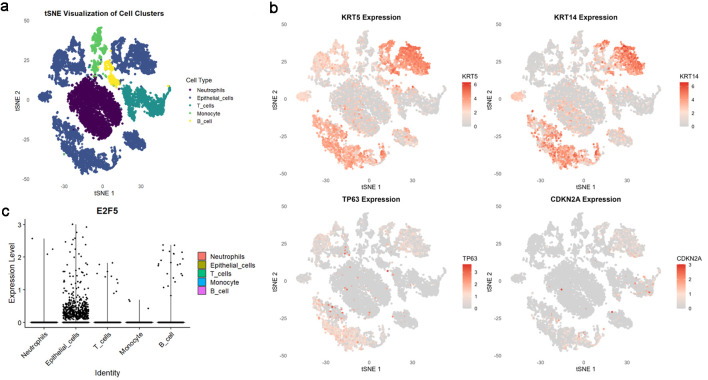
High E2F5 expression in LSCC cells. (a) tSNE plot showing the distribution of different cell types in the single-cell population. (b) LSCC cells labeled with KRT5, KRT14, TP63, and CDKN2A. (c) Distribution of E2F5 expression in single-cell RNA sequencing data from LSCC. LSCC: laryngeal squamous cell carcinoma.

**Figure 2:**
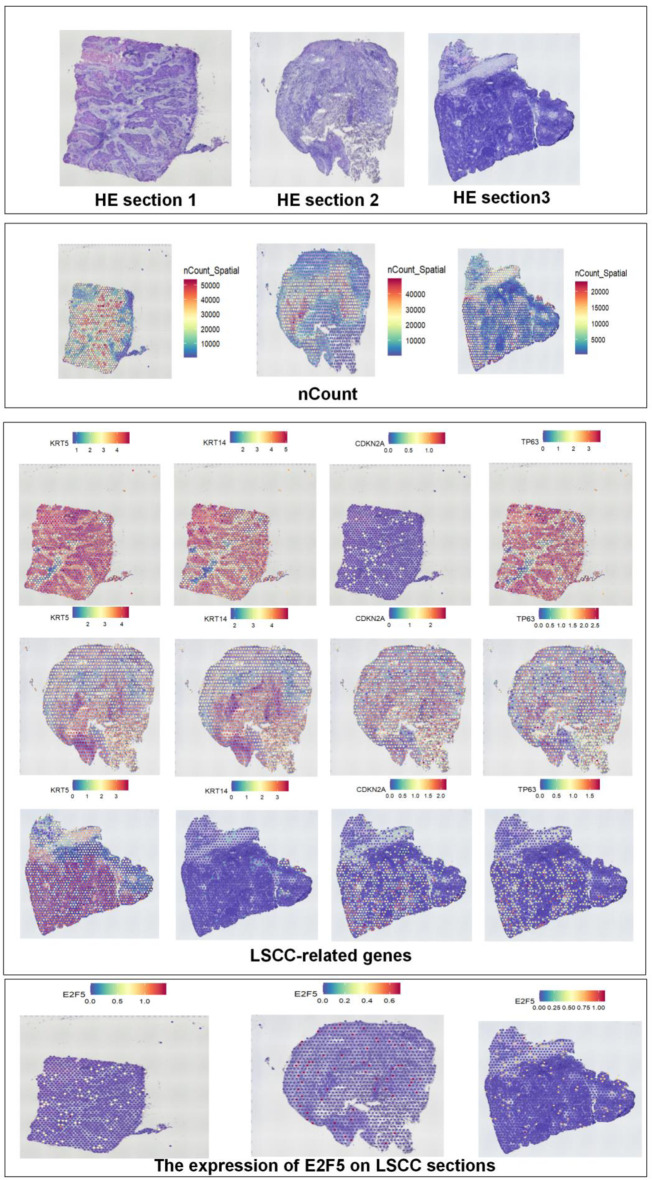
Spatial transcriptomics data confirm high expression of E2F5 in LSCC tissue. Violin plot of spatial counts, showing gene expression distribution in LSCC samples, accompanied by the corresponding spatial heat map. Spatial expression map of E2F5 in LSCC tissue for samples. LSCC: laryngeal squamous cell carcinoma.

**Figure 3:**
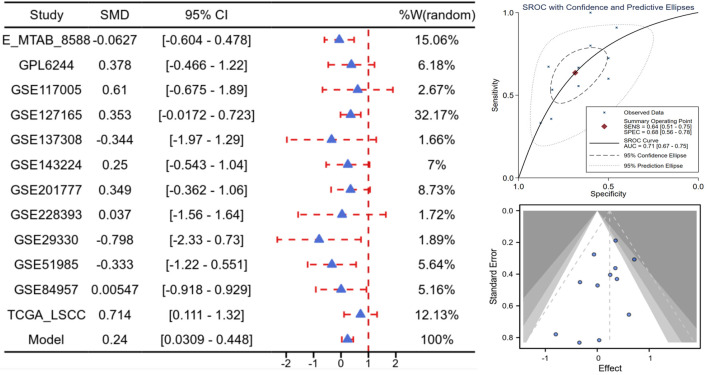
High E2F5 expression in LSCC at the mRNA level. Forest plot of SMD and their 95% CI, showing the means, SD, and SMD for each study’s experimental and control groups. Funnel plot from the Begg test to assess publication bias across studies. sROC curve, presenting the evaluation of discrimination ability. CI: confidence interval; LSCC: laryngeal squamous cell carcinoma; SMD: standardized mean difference; sROC: summary receiver operating characteristic.

**Figure 4:**
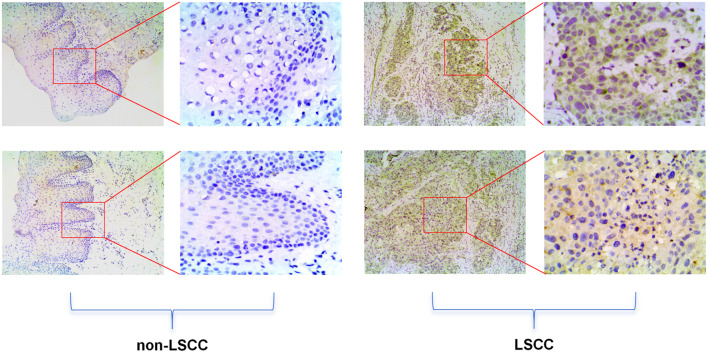
High E2F5 expression in LSCC tissues at the protein level. The top panel shows immunohistochemical staining of non-LSCC tissues, and the bottom panel shows immunohistochemical staining of LSCC tissues. LSCC: laryngeal squamous cell carcinoma.

**Figure 5:**
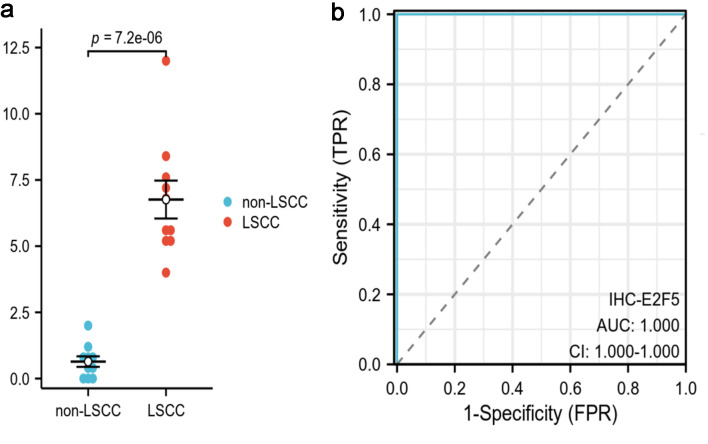
High E2F5 expression in LSCC tissues at the protein level, as determined by staining counts. (a) Differential expression honeycomb plot. (b) ROC curve. LSCC: laryngeal squamous cell carcinoma; ROC: receiver operating characteristic.

Based on scRNA-seq, ST, and mRNA-level analyses, E2F5 exhibited significant high expression in LSCC epithelial cells (Fig. 1c). Concurrently, LSCC-related markers KRT5, KRT14, TP63, and CDKN2A also showed high expression in these epithelial cells (Fig. 1a, b). ST analysis further confirmed sustained high E2F5 expression in LSCC tissues, with LSCC-related genes KRT5, KRT14, TP63, and CDKN2A similarly exhibiting high expression at the tissue level (Fig. 2). mRNA-level analysis revealed significant E2F5 overexpression, with SMD = 0.24, 95% CI = 0.0309 - 0.448, sROC AUC = 0.71, and no significant heterogeneity among studies (Fig. 3). IHC staining of clinical samples further validated high E2F5 expression in LSCC tissues (Fig. 4), with P = 7.2 × 10^-6^ and ROC AUC = 1 (Fig. 5).

### E2F5 knockdown inhibits LSCC-related cell growth

**Figure 6:**
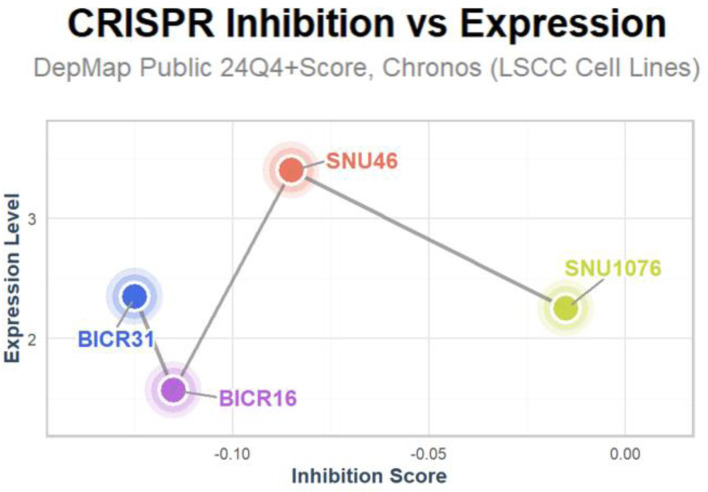
Inhibition of LSCC-related cell lines upon E2F5 knockdown. LSCC: laryngeal squamous cell carcinoma.

E2F5 was found to be highly expressed in LSCC-related cell lines BICR31, BICR16, SNU46, and SNU1076. Upon gene knockdown to reduce E2F5 expression, growth in these cell lines was significantly inhibited, with inhibition score < 0 (Fig. 6).

### High E2F5 expression alters immune cell enrichment

**Figure 7:**
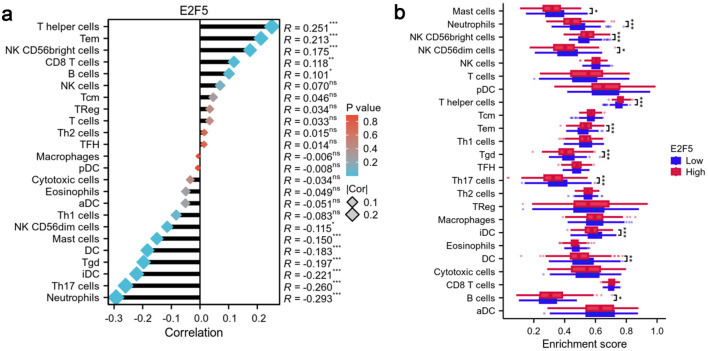
High expression of E2F5 in LSCC affects the enrichment status of immune cells. (a) Correlation between E2F5 expression and immune cells. (b) Differences in immune cells between high and low E2F5 expression groups. Significance symbol: *P < 0.05, **P < 0.01, ***P < 0.001. LSCC: laryngeal squamous cell carcinoma.

Correlation analysis indicated that T-helper cells, effector memory T cells, and natural killer (NK) CD56bright cells exhibited the strongest positive correlations with E2F5 expression (R = 0.251, 0.213, and 0.175, respectively). Conversely, neutrophils, Th17 cells, and immature dendritic cells (DCs) showed significant negative correlations with E2F5 expression (R = -0.293, -0.260, and -0.221, respectively) (Fig. 7a). Comparison of immune cell enrichment scores between high and low E2F5 expression groups revealed significant differences in the immune microenvironment. In the high E2F5 expression group, T-helper cells, effector memory T cells, and NK CD56bright cells showed significantly higher enrichment compared to the low expression group, while neutrophils, Th17 cells, and immature DCs exhibited significantly lower enrichment in the high E2F5 expression group (Fig. 7b).

### E2F5 may influence LSCC via NETs

**Figure 8:**
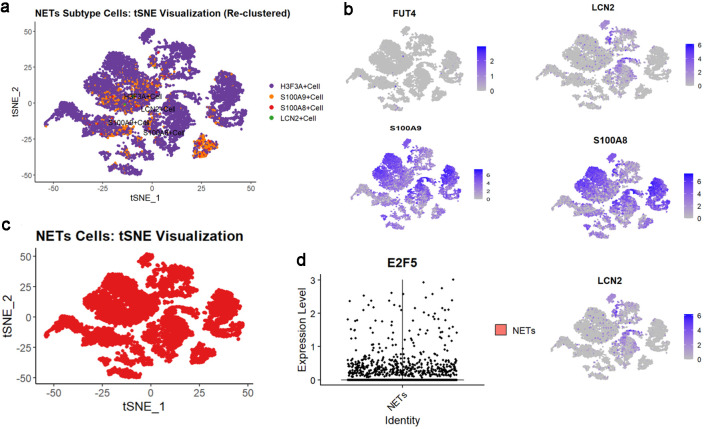
High expression of E2F5 in NETs in LSCC single-cell RNA sequencing. (a) tSNE subtype plot of NETs expression in LSCC. (b) Expression plot of NETs-related indicators. (c) Overall tSNE plot of NETs expression in LSCC. (d) Expression of E2F5 in NETs. LSCC: laryngeal squamous cell carcinoma; NETs: neutrophil extracellular traps.

**Figure 9:**
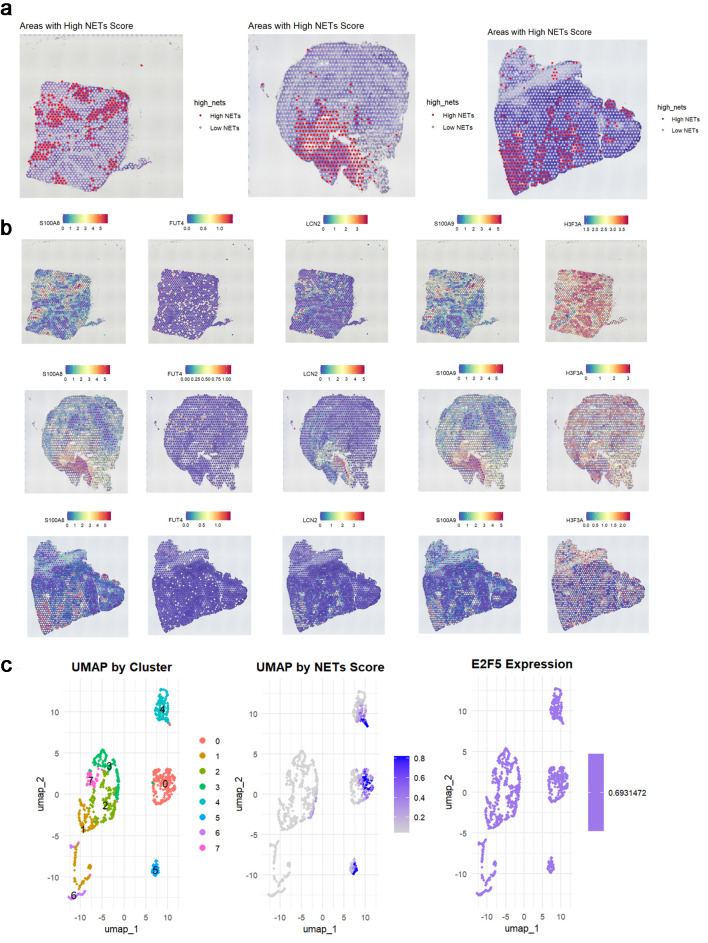
E2F5 expression in LSCC is associated with neutrophil extracellular traps. (a) Spatial distribution of high and low NETs scores across three tumor samples. (b) Spatial expression patterns of key NET-associated markers (S100A8, FUT4, LCN2, S100A9, HSP3A) in corresponding tissue sections. (c) UMAP visualization showing cell clusters, NETs score distribution, and E2F5 expression pattern in single-cell data. LSCC: laryngeal squamous cell carcinoma; NETs: neutrophil extracellular traps.

**Figure 10:**
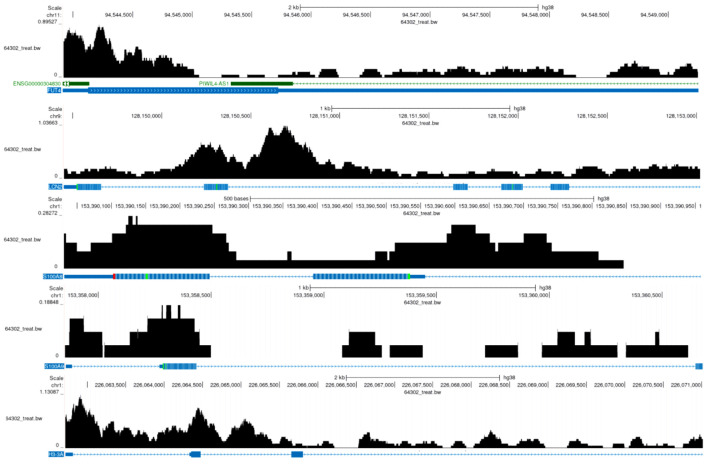
E2F5 transcription factor in NETs-related regions. The black histogram above represents sequencing read depth (64302_treat_bw), the middle colored bands indicate predicted functional elements, and the blue markers below show RefSeq predicted gene models. The gene regions of FUT4, LCN2, S100A8, S100A9, and H3-3A display multiple binding peaks. NETs: neutrophil extracellular traps.

Based on the expression patterns of NETs-related markers (S100A8, FUT4, LCN2, S100A9, and HSP3A), NETs-related cell subpopulations were identified (Fig. 8a-c). Analysis revealed significant E2F5 enrichment in NETs high-expression cells (Fig. 8d). ST analysis showed widespread NETs distribution in tumor tissues (Fig. 9a), with NETs-related markers S100A8, FUT4, LCN2, S100A9, and HSP3A exhibiting high expression across tissue regions (Fig. 9b). Integration and dimensionality reduction of data from three tissue sections confirmed high E2F5 expression in NETs-enriched regions (Fig. 9c). Further ChIP-seq analysis revealed multiple binding peaks for E2F5, as a transcription factor, in the gene regions of S100A8, FUT4, LCN2, S100A9, and HSP3A (Fig. 10).

**Figure 11:**
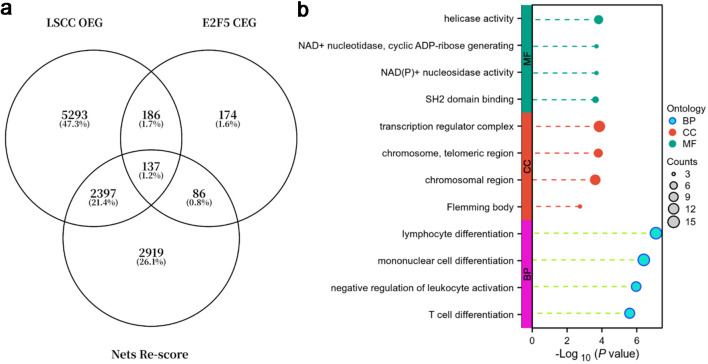
E2F5 and NETs-related genes are primarily enriched in immune regulation and chromosome/transcription regulation. (a) Venn diagram of LSCC overexpressed genes (OEGs), E2F5 co-expressed genes (CEGs), and genes with NETs-related scores > 1.5. (b) Gene function enrichment analysis. LSCC: laryngeal squamous cell carcinoma; NETs: neutrophil extracellular traps.

Additionally, analysis of E2F5 co-expressed genes, LSCC overexpressed genes, and genes with NETs-related scores > 1.5 (Fig. 11a) showed that these genes were primarily enriched in the following functional pathways: in biological process (BP), they were mainly involved in lymphocyte differentiation, monocyte differentiation, negative regulation of leukocyte activation, and T-cell differentiation; in cellular component (CC), they were primarily associated with transcriptional regulatory complexes, telomeric regions, chromosomal regions, and Flemming bodies; in molecular function (MF), they were mainly linked to helicase activity, NAD^+^ nucleosidase/ADP-ribose generation activity, NAD(P)^+^ nucleosidase activity, and SH2 domain binding (Fig. 11b).

## Discussion

This study, through integrating scRNA-seq, ST, mRNA expression profiling, and IHC validation, systematically revealed the high expression characteristics of E2F5 in LSCC and its potential association with NETs, providing new perspectives for elucidating the molecular mechanisms and tumor microenvironment regulation in LSCC. As a member of the E2F family, E2F5 plays a significant role in transcriptional regulation, but its function in LSCC remains unclear. Utilizing CRISPR knockout screening combined with the CERES algorithm, this study found that E2F5 exhibits significant dependency in LSCC cell line proliferation. Negative dependency scores indicated that E2F5 knockout suppresses cell growth, supporting a consistent pro-tumorigenic role for E2F5 in LSCC. This dual role provides clues to the complex functions of E2F5 in LSCC, warranting further investigation into its molecular mechanisms. E2F5 showed significant high expression in LSCC epithelial cells and NETs-related cells, suggesting that it not only drives tumor cell proliferation but may also participate in LSCC progression by regulating microenvironment-related genes. Notably, high E2F5 expression was strongly correlated with the enrichment of NETs hallmark genes (e.g., S100A8, S100A9, LCN2), and ChIP-seq analysis revealed E2F5 binding peaks in the regulatory regions of these genes, suggesting that E2F5 may indirectly influence NETs-related gene expression as a transcription factor. This finding provides critical evidence for E2F5’s role in the LSCC microenvironment, filling a gap in related research.

High E2F5 expression may significantly reshape the LSCC microenvironment by regulating NETs formation. NETs, released by neutrophils via NETosis, are enriched with DNA, histones, and molecules such as S100A8, S100A9, and LCN2, playing roles not only in antibacterial defense but also in supporting tumor progression by releasing pro-inflammatory factors, inducing immune suppression, and promoting angiogenesis. This study found significant E2F5 enrichment in NETs high-expression regions, with NETs hallmark genes broadly upregulated in LSCC tissues, suggesting that E2F5 may enhance NETs formation by transcriptionally regulating genes like S100A8/A9 and LCN2. This mechanism aligns with prior studies, such as those showing S100A8/A9 amplifying inflammatory responses via ROS and PAD4-mediated NETs formation [11]. In LSCC, E2F5 may exacerbate tumor-associated inflammation and immune evasion by activating NETs-related signaling pathways (e.g., AMPK/Nrf2/HO-1) [11, 19]. ST results further validated high E2F5 expression in NETs-enriched regions, suggesting that NETs may act as an “amplifier” of E2F5-mediated tumor-promoting signals, promoting LSCC invasion and metastasis by locally releasing pro-inflammatory factors and establishing an immunosuppressive microenvironment. Additionally, the critical role of LCN2 in NETs [12] suggests that E2F5 may further enhance NETs’ tumor-promoting effects by regulating LCN2.

Functional enrichment analysis of E2F5 co-expressed genes, LSCC overexpressed genes, and NETs-related genes revealed E2F5’s molecular mechanisms in LSCC. In CC, these genes were primarily enriched in transcriptional regulatory complexes, telomeric regions, chromosomal regions, and Flemming bodies, with studies indicating that Flemming body enrichment may support rapid LSCC cell proliferation by regulating AURKB [20]. This suggests that E2F5 may drive tumor progression by regulating gene transcription and chromatin organization. For instance, E2F5 may interact with the RNA Pol II complex to upregulate tumor-promoting genes or promote cell immortalization by regulating TRF1 [21-23]. In MF, E2F5-related genes were associated with helicase activity, NAD^+^ nucleosidase/ADP-ribose generation activity, NAD(P)^+^ nucleosidase activity, and SH2 domain binding, indicating that E2F5 may support tumor cell proliferation and survival by regulating DNA unwinding (e.g., MCM proteins [24]), DNA damage repair (e.g., PARP1 [25]), and signal transduction (e.g., PI3K/AKT [26]). These functions may be linked to NETs formation, with NAD^+^ nucleosidase activity providing metabolic support for NETosis [27] and SH2 domain binding potentially activating pro-inflammatory signaling pathways [28], enhancing NETs’ tumor-promoting effects. E2F5 co-expressed genes were also involved in lymphocyte differentiation and negative regulation of leukocyte activation, suggesting that E2F5 may suppress anti-tumor immunity via SH2 domain binding [29].

E2F5’s interactions with the immune microenvironment underscore its multifaceted roles. ssGSEA analysis showed that high E2F5 expression was positively correlated with Th cell, effector memory T cell, and NK CD56bright cell enrichment, but negatively correlated with neutrophils, Th17 cells, and immature DCs. These immune cell distribution differences may stem from E2F5’s regulation of the microenvironment via NETs. NETs can suppress anti-tumor immunity by releasing pro-inflammatory factors like interleukin (IL)-8 [30] while promoting myeloid-derived suppressor cell enrichment [31]. In LSCC, E2F5 may inhibit CD8^+^ T-cell activity [32] and promote immune evasion by enhancing NETs formation. Low neutrophil enrichment may be related to NETosis depletion [33]. Notably, the association between high E2F5/NETs signatures and reduced neutrophil infiltration supports a model in which persistent NETosis exhausts local neutrophils, positioning neutrophil depletion as a central mechanism rather than a secondary correlate. Enhanced NAD^+^ nucleosidase activity may further support the metabolic demands of this E2F5-NETs axis, while enhanced NAD^+^ nucleosidase activity supports the E2F5-NETs axis in energy metabolism. Enrichment of E2F5-related genes in transcriptional regulatory complexes suggests that E2F5 may regulate immunosuppressive factor expression, such as TGF-β, reinforcing an immunosuppressive microenvironment.

Despite providing significant evidence, this study has limitations. Direct regulation of NETs hallmark genes by E2F5 requires validation via ChIP-qPCR, and the mechanisms of E2F5-NETs signaling pathways (e.g., AMPK/Nrf2/HO-1) need further exploration. E2F5’s dual roles in different cell lines may be linked to molecular subtypes (e.g., TP53 mutations), requiring multi-omics analysis. As this study was based on *in vitro* cell lines and tissue samples, the functions of E2F5 and NETs in LSCC *in vivo* models require animal studies for validation. Future research should explore the therapeutic potential of E2F5 inhibitors or NETs-targeted drugs (e.g., Paquinimod), which may improve LSCC prognosis when combined with immune checkpoint inhibitor (ICI). For instance, Paquinimod significantly reduces inflammation by inhibiting S100A8/A9 [11], suggesting its potential application in LSCC.

### Conclusion

In summary, this study is the first to reveal E2F5’s high expression in LSCC and its potential interaction with NETs, demonstrating that E2F5 may promote tumor proliferation and microenvironment remodeling through transcriptional regulation, chromatin organization, and NAD^+^-related functions. The identification of the E2F5-NETs axis suggests a possible therapeutic avenue for LSCC, but further *in vivo* and functional studies are required to substantiate its clinical relevance.

## Data Availability

The data supporting the findings of this study are available from the corresponding authors upon reasonable request.
